# Editorial: Genetic response and resistance in plants towards abiotic and biotic stresses

**DOI:** 10.3389/fpls.2024.1505006

**Published:** 2024-12-10

**Authors:** Sushil Satish Chhapekar, Sachin Gorakshnath Chavan

**Affiliations:** ^1^ Division of Plant Science and Technology, University of Missouri, Columbia, MO, United States; ^2^ Hawkesbury Institute for the Environment, Western Sydney University, Richmond, NSW, Australia

**Keywords:** plant stress, biotic stress, abiotic stress, genetic response, tolerance, resistance

## Introduction

Plants worldwide are increasingly facing severe abiotic stresses such as extreme temperatures, salinity, nutrient deficiency and drought, as well as biotic stresses including damages from pests, pathogens (bacteria, viruses, fungi) and herbivores. These challenges are worsened by climate change, leading to ecological damage and food insecurity. Plants respond to both biotic and abiotic stresses through various mechanisms. These include molecular, cellular, and physiological changes, such as activating stress-responsive genes, producing protective proteins, and modifying hormone levels. These responses help plants mitigate damage, maintain growth, survive and adapt to adverse conditions. Understanding these responses is critical for developing stress-resistant crop varieties that can thrive in challenging environments. This Research Topic explores the genetic responses and resistance mechanisms that plants employ to withstand such stresses. The topic aims to synthesise knowledge on the genetic responses of various plant and crop species and their adaptations to biotic and abiotic stresses. The Research Topic includes 23 studies (19 research and 4 review articles) on natural genetic evolution, gene manipulation, and plant physiological and biochemical changes during stress response. Together, these research contributions advance our understanding of plant resilience and offer critical insights for sustainable agriculture and future research directions. The articles can be broadly categorised into plant responses to general (both biotic or abiotic), biotic or abiotic stress.

## General stress responses in plants

Plant responses could be specific to biotic or abiotic stress and can also be triggered by both biotic and abiotic stress which could be categorised under general stress response. The two review articles by Wang et al. and Sun et al. meticulously discuss the roles of uncharacterised genes and actin-depolymerizing factor (ADF) in plant general stress response, respectively.

Wang et al. provided a comprehensive review of the role of uncharacterised genes in plant development and stress responses. The review summarizes the present knowledge of these genes encompassing their structures, classifications, and functions in plant growth and stress resistance. In addition, there has been progress in the techniques used to uncover the roles of these genes. Discovering the molecular functions of previously uncharacterised genes could reveal strategies to optimize key physiological processes in plants. This would enhance the functional network system of plants and offer more opportunities for adaptive improvement. Sun et al. reviewed “the roles of actin-depolymerizing factor (ADF) in plant stress responses”. This review provides an overview of the developments in plant *ADF* gene research. They concluded that (a) the plant *ADF* family genes could be explored across a broader spectrum to enhance plant stress resistance (b) the analysis of *ADF* gene regulation in stress resistance of plants and manipulation of *ADF* genes through genome-editing techniques are crucial avenues for future investigation in this field.

## Plant responses to biotic stresses

Among several biotic stresses, the blast disease severely affects plant growth and crop production in rice. Overexpression of *OsSRDP* (*Oryza sativa* Stress Responsive DUF740 Protein) gene under AtRd29A promoter showed tolerance to rice blast (*Magnaporthe oryzae*) disease (Jayaraman et al.). Interestingly, these rice transgenic plants were significantly tolerant to abiotic stress including drought, salinity and cold demonstrated by physiological, biochemical and molecular assays. The novel *OsSRDP* gene identified in this study indicates the importance and functionality of DUF740 family gene in multiple stress tolerance in rice. In potatoes, late blight (*Phytophtora infestans*) is a major disease that leads to serious yield loss. Forty potato genotypes were screened for late blight resistance using an assay which identified five highly resistant wild species including PIN45 (*S. pinnatisectum*), CPH62 (*S. cardiophyllum*), JAM07 (*S. jamesii*), MCD24 (*S. microdontum*), and PLD47 (*S. polyadenium*) (Bhatia et al.). Transcriptome sequencing of these lines demonstrated several key candidate genes regulating late blight resistance and multiple signalling pathways. Differential gene expression (DGE) and gene ontology (GO) analysis revealed significantly important genes for stress-response, starch metabolism transcription factors, photosynthesis and phytohormones pathway. This research underscores the potential of DUF740 family genes for breeding rice varieties with enhanced tolerance to multiple stresses.

Stripe rust induced by *Puccinia striiformis* (*Pst*), is usually considered a major constraint for wheat production globally. Genome-wide transcriptome profiles in two wild emmer wheat genotypes with contrasting degree of resistance to (Pst) demonstrated the presence of key transcription factors (WRKY, AP2-ERF, bZIP and MADs), and pathways associated with the response and regulation of *Pst* infection in wheat (Ren et al.). These research findings contribute to understanding the molecular mechanisms of the response to strip rust.

Recently, aphid has become an emerging and most devastating pest of sorghum. A combination of transcriptomic and pathway analyses of sorghum genotypes (resistant Tx2783 and susceptible BTx623) demonstrated key transcription hubs and pathways associated with aphid response (Shrestha et al.). This study meticulously identified several genes associated with signal perception (NBS-LRR), signal transduction MAP kinases, salicylic acid and jasmonic acid pathways which shed light into the understanding of the molecular mechanism of host-pathogen interactions.

In addition to biotic challenges, plants also face several abiotic threats, including growth environment and nutrient conditions, which are described further.

## Plant responses to abiotic stresses

The abiotic stress category included plant response to drought, salinity, heat stress, nutrient deficiency and toxic element accumulation. Rai et al. reviewed the role of WRKY proteins in plant salinity stress. The study provides a comprehensive examination of how salinity affects plants globally, emphasizing the critical need to address this issue for food security. The study delves into the mechanisms by which plants detect and respond to salt stress, highlighting the role of WRKY proteins in this process. WRKY proteins are key regulators that modulate various plant responses to salinity, helping plants to better cope with and survive in high-salinity environments.

Graci et al. reviewed plant response to heat stress in tomatoes, particularly during the reproductive stage which has a direct impact on yield. The review covers a wide range of genes and their roles in helping plants cope with high-temperature conditions, which is crucial for maintaining yield. The key genes include (a) transcriptional factors (TFs): TFs regulate the expression of other genes, helping plants respond to heat stress by activating or repressing specific pathways; (b) heat shock proteins (HSPs): HSPs act as molecular chaperones, protecting other proteins from denaturation and aggregation caused by high temperatures; (c) genes related to flowering and fruit set: these genes ensure the proper development of flowers, pollen, and fruit, which are crucial for plant reproduction and yield, even under heat stress; (d) epigenetic mechanisms (DNA methylation, histone modification, chromatin remodelling, and non-coding RNAs).

Interactive abiotic climate change factors like atmospheric CO_2_, temperature and water have complex effects on plant growth and survival. Opoku et al. investigated diverse physiological and yield responses of barley (C3 crop) and sorghum (C4 crop) to different climatic conditions and interactions. This complex association indicates sorghum is more vulnerable to climate change compared to relatively more stable barley. Furthermore, under some conditions with the combination of high CO_2_ and temperature, with enough available water, C4 crops may outperform. In contrast, the C3 crops perform better under drought stress combined with lower temperature conditions.

Functional characterisation of stress-responsive genes using transgenic plants is a well-established approach to comprehending their critical role in climate resiliency and further exploiting them for crop improvement program. Zhu et al. showed that a ‘mulberry 9-*cis*-epoxycarotenoid dioxygenase’ (*MaNCED1)* gene is actively involved in the regulation of plant growth and imparts tolerance to drought and salt in transgenic tobacco. They also suggested that abscisic acid coordinates with ethylene and auxin to control the seed germination, abiotic stress tolerance and seed growth. Sun et al. showed that *Glycine max* actin-depolymerizing factor (*GmADF*) genes are involved in diverse abiotic stresses in particular drought and salt stress in soybean. Segmental duplication is majorly associated with *GmADF* gene expansion during evolution. The potential candidate genes need to be tested and studied through functional studies for soybean improvement. Sun et al. investigated the ‘plant AT-rich sequences and zinc-binding proteins’ (PLATZ) gene family (that are significantly involved in environmental stress response) in Rosaceae species including apple, peach, pear and strawberry. Genome-wide identification analysis demonstrated 104 PLATZ genes in the apple genome. Expression, co-expression network and subcellular localization demonstrated specific MdPLATZ genes are majorly involved in drought and ABA stress. Chen et al. performed integrated metabolomic, transcriptomic, and physiological analysis of the effect of silicon dioxide nanoparticles on *Ehretia macrophylla* seedlings during severe drought stress. The differentially expressed genes (DEGs) regulated by silicon particles are mainly involved in pathways of auxin signal transduction and protein kinase signalling. They demonstrated that metabolism of α-linolenic acids and fatty acids plays an active role in improvement of drought tolerance in *E. macrophylla* plants.

Plant stress is generally regulated by multiple traits. However, most of the research studies are typically associated with single traits/genes. Genome-wide association studies (GWAS) emerged as an effective method that studies complex or multiple traits associated to single/multiple stress. In alfalfa, (*Medicago sativa*) Mnafgui et al. performed comprehensive GWAS for the traits related to biomass and growth of 129 accessions. A combined stress of salinity (abiotic) and *Phoma medicaginis* (biotic) infection was given to these genotypes and GWAS was carried out to characterize the significant association of SNPs and 7 growth traits. Key significant SNPs were identified for the number of healthy leaves, chlorotic leaves, length of the main stem, aerial weight (fresh & dry) and necrotic leaves. The study reveals significant markers for traits associated to cellular metabolism, cell wall rigidity and NBS-LRR gene family. Altogether, these analyses identified 5 alfalfa accessions tolerant to combined stress which could be important for the alfalfa breeding program. Sharma et al. conducted genome-wide identification and transcriptome-based expression profiling of the *NHX* gene family in wheat during salt stress. They are unevenly distributed across 18 chromosomes, with none on chromosomes 6A, 6B, and 6D. Segmental duplication events were crucial for the expansion of these genes. Phylogeny, protein-protein interactions, promoter and qRT-PCR analysis showed that key TaNHX genes are associated with salt stress tolerance in wheat. This research highlights the importance of the wheat NHX family in response to salt stress and provides a basis for further investigation. In another excellent genome-wide analysis by Zhong et al. in peanuts under abiotic stress and hormone treatments, regulatory mechanisms and expression profiles of *TGA* genes were revealed. The study identifies 20 genes classified into five groups. These genes showed different expression patterns in low temperature, drought stress and in response to hormonal stress. Among all the genes, *AhTGA11* was functionally validated in *Arabidopsis* transgenic and hairy roots of transgenic soybean, demonstrating their significant involvement in low temperature, as well as drought by regulating jasmonic acid, salicylic acid and abscisic acid. These findings offer valuable insights for breeding abiotic-resistant peanut varieties.

In wheat, Saroha et al. identified heat-responsive micro-RNAs (miRNA) in contrasting genotypes during the reproductive stage under heat stress. Several miRNAs showed genotype-specific expression patterns while many differentially expressed novel miRNAs were identified under heat stress (HS). Degradome analysis clearly demonstrated the several hundred targets of miRNAs in heat stress indicating the vital role played by miRNA for HS tolerance. Heat stress modifies the plant transcriptome at the transcription level as well as alters alternative splicing (AS), and intron retention (IR). He et al. showed that in response to HS, the *Arabidopsis* plant showed a reduction in the expression of the NTC-related protein 1 *-* associated complex to provide the requirement for transcription of heat-responsive genes like heat shock proteins (HSPs) and heat shock factors (HSFs). These genes help to accumulate improperly spliced especially IR products and eventually cause harm to plants. TS et al. carried out a comprehensive genetic analysis of heat tolerance in hot pepper using QTL mapping. They developed a biparental F_2_ mapping population by crossing the heat tolerant (DLS-161-1) and heat susceptible (DChBL-240) genotypes which exhibit contrasting phenotypic differences for heat tolerance to characterise the genetic architecture underlying heat tolerance in hot pepper. Many QTLs for physiological traits, fruit yield per plant, chlorophyll content, morphological traits (including plant height) for heat tolerance were identified in this study. Further, fine mapping of these QTLs and new markers developed from these loci will offer better insights for the development of heat-tolerant varieties. Another heat-stress response study by Chaddad et al. demonstrated a meta-analysis of microarray data in *A*. *thaliana* under heat stress. They identified 1972 significant DEGs associated with heat tolerance specifically associated with pathways for heat-shock protein, mRNA splicing, unfolded protein binding and secondary metabolite biosynthesis. One of the key insights of the study is that heat stress affects the expression of genes not only at the transcriptional level but also at the post-transcriptional level.

The abiotic stress category also included studies on iron deficiency chlorosis (IDC), phosphorus use efficiency (PUE) and cadmium (Cd) accumulation. Kohlhase et al. showed that the QTL on soybean chromosome Gm03 is comprised of four unique linkage blocks, with every single block contains certain candidate genes for IDC. They validated the function of 3 candidate genes through virus-induced gene silencing by developing 3 single-gene constructs to target *GmGLU1* (*Glutamate Synthase*, Glyma.03G128300), *GmRR4* (*Response Regulator*, Glyma.03G130000), and *GmbHLH38* (*beta Helix Loop Helix*, Glyma.03G130400 and Glyma.03G130600). Results showed that *GmRR4* and *GmGLU1* silenced plants affect the iron uptake/transport ability that leads to iron deficiency chlorosis. This study demonstrated the importance of the Gm03 QTL region and the need for it to be investigated in detail to validate the underlying candidate genes for IDC in soybean and related legume crops. Zeffa et al. performed a multi-locus GWAS for phosphorus use efficiency (PUE) in 132 maize inbred lines. The study identified 306 quantitative trait nucleotides (QTNs) associated with the 24 agronomic traits (including roots, shoots, plant height, phosphorus utilization and contents) and 186 potential candidate genes significantly associated with various molecular functions such as transcription regulators and factors, transporters, and transferase. In summary, the identified QTNs, favourable alleles and genes provide new insights into the genetic architecture of PUE and to develop better maize varieties. Li et al. investigated the mechanism of cadmium (Cd) accumulation by *Sedum plumbizincicola* where they demonstrated a novel protein associated to cadmium stress by using comparative transcriptome and iTRAQ proteome analysis. Altogether, the results illustrate that the newly identified protein *Sedum plumbizincicola* SIZ1 (SpSIZ1) showed interactions with SpABI5, that increase the expression of downstream genes of abscisic acid signalling pathway which helps for Cd tolerance.

## Conclusions

The studies presented in this Research Topic highlight the diverse genetic, molecular and physiological mechanisms plants employ to counteract abiotic and biotic stresses. The identified stress tolerance mechanisms, novel QTLs/genes, and advanced methods and approaches described in these articles facilitate the knowledge and provide resources and future directions for crop improvement. Altogether, these advancements help to develop climate-smart crops with better productivity and climate resilience that plays a major role to ensure food security worldwide during climate change.

